# Older adult perspectives on emotion and stigma in social robots

**DOI:** 10.3389/fpsyt.2022.1051750

**Published:** 2023-01-12

**Authors:** Jill A. Dosso, Jaya N. Kailley, Gabriella K. Guerra, Julie M. Robillard

**Affiliations:** ^1^Division of Neurology, Department of Medicine, The University of British Columbia, Vancouver, BC, Canada; ^2^British Columbia Children’s and Women’s Hospital, Vancouver, BC, Canada

**Keywords:** social robots, older adults, dementia, care partners, co-creation, emotional alignment, stigma

## Abstract

**Introduction:**

Social robot adoption by older adults and people living with dementia is limited by several barriers, including a lack of emotional alignment with social robots and perceptions of stigma around social robot use. The goal of this work was to address these barriers by engaging end-users in discussions on factors that could affect emotional connection to a social robot and considerations around using a social robot in public.

**Methods:**

We conducted seven 90-min online workshops with older adults with (*n* = 2) and without dementia (*n* = 25) and care partners (*n* = 17). Older adults with and without dementia were in their 50s – 80s, and care partners were in their 30s – 70s. Seven older adults, seven care partners, and one older adult with dementia indicated that they had used a robot before. Online workshops had 4-8 participants each, and began with video demonstrations of pet-like robot MiRo and tabletop robot T-Top, as well as a live demonstration of MiRo. Participants completed the Multi-Dimensional Robot Attitude Scale before the workshops and the Psychosocial Impact of Assistive Devices Scale and two Zoom polls during the workshops. The audio and chat responses from the workshops were transcribed verbatim and content analysis was performed on the transcripts.

**Results:**

Our analysis revealed three broad themes and 10 subthemes. In their discussions on features and applications, participants highlighted preferred forms of communication with a robot and ways in which a robot could support connection between people. For example, robots could improve the quality of communication between care partners and the person for whom they care. While many agreed that a social robot should match their level of emotion and interactivity, participants had different preferences for social robot emotional range and display features. Finally, participants discussed considerations around showing a robot to other people; several participants suggested that a robot could help raise awareness of ageing and dementia while others shared concerns about stigma and attracting negative attention from an audience.

**Discussion:**

Incorporating these findings into the design and implementation of social robots will result in devices that are better-suited to the needs of older adults, people living with dementia, and care partners.

## 1. Introduction

Maintaining independence and quality of life are key priorities for many of Canada’s 7.1 million older adults (1) including over 500,000 individuals currently living with dementia and their family members (2). These goals may be supported by complementing human care with robotic assistive technologies. Social robots are defined as devices that can assist a person through interaction and include humanoid robots such as Pepper (3) and Nao (4), pet-like robots such as PARO (5) and MiRo (6), and avatar-based devices (7).

Social robots have numerous functions and uses. They can engage in conversation with the user, recognise people, and identify basic emotions (3). Social robots can also be sources of entertainment (e.g., by playing music and providing interesting facts) (8), and activities of daily living may be supported by social robot functionalities such as reminders and timers (8, 9). These emerging devices are increasingly complex; they can monitor the user’s health, facilitate video calls, provide cognitive training activities (10), and assist with physical activity (11). Such robots show promise in supporting ageing in place and promoting the cognitive health of older adults. For example, a recent scoping review revealed that social robots may support persons living with dementia by decreasing behavioural and psychological symptoms, facilitating social interaction, and improving mood (12). Interaction with social robots has also been shown to decrease loneliness, blood pressure, pulse rate, and support pain management and behavioural medication use (13–15).

The ongoing COVID-19 public health crisis and resulting public health policies – including social distancing measures and restrictions on in-person contact – are already having important measurable impacts on older adults’ health and mental health; persons living with dementia have reported an increase in stress and feelings of isolation due to the pandemic (16). Social robots have the potential to alleviate loneliness by providing companionship when in-person contact is not possible. Since the onset of the pandemic, social robot implementation has accelerated. For example, in May 2022, New York State’s Office for the Ageing purchased over 800 domestic robots for older adults (17). As a result, it is important to ensure that these emerging solutions are aligned with the goals and values of end-users and are supported by high-quality evidence of their effectiveness. Previous research has shown that older adults and roboticists have very different preferences for the design of social robots (18), highlighting the importance of engaging end-users in social robot development to ensure that devices are well-suited to their unique needs and priorities.

Increasingly, researchers are working directly with older adults and people living with dementia to find out their priorities around potential social robot interventions. Older adults have expressed preferences for a social robot that can provide companionship, interact, help with chores, and call for help in emergencies (19). They have also reported that they would like social robots to help with certain tasks like communication and medication reminders, but not others like bathing, toileting, and managing finances (20). Social robot concerns raised by older adults include the potential for a robot to become a tripping hazard or replace connection with other people or pets (19).

The needs and priorities of older adults living with dementia and their care partners are less well-described. In one of our previous studies, care partners were more enthusiastic about using robots compared to older adults with and without dementia, and older adults with and without dementia expressed preference for a mobile robot over a static robot (19). Another study found that care staff, care home residents and their family members desired a robot with a high degree of interactivity, emotional display, and the ability to act on command (21). It is important to include older adults with and without dementia in the process of social robot development as their thoughts around social robot design and application areas may differ (18).

Different types of social robots can raise distinct ethical considerations. For example, the existing literature has discussed the potential for deception (10, 22, 23) or loss of dignity (24) upon usage of emotional humanoid or pet-like social robots by older adults. At the same time, older adults often express preference for realistic social robots over toy-like, unfamiliar designs (19, 21), making deception a complex ethical issue in social robot development.

As the research above reveals, end-users can provide valuable insights into aspects of social robot development and implementation such as features, applications, barriers to adoption, and ethical issues. In the present work, we focus on another key consideration for social robot adoption and sustained use: emotional alignment between user and robot, and the implications of that alignment for perceptions of stigma.

A lack of emotional alignment between social robots and older adults has recently been recognised as a barrier to social robot adoption (25, 26). Emotional alignment between two interaction partners occurs when one partner aligns their emotions with the other partner’s emotions, for example, during an empathic response (27). In the context of this study, emotional alignment is studied in the context of human–robot interactions. In order to improve social robot communication abilities, researchers and technology developers are working to build robots that can interpret a user’s emotional state and display emotional states of their own (27, 28). People may form emotional connections with robots, even those that are not socially assistive, such as home-cleaning robots (29). Further, previous research with older adults suggests that the ability of social robots to be interactive, responsive, and display empathy could increase human–robot emotional connection (18, 24, 30–32). Our recent work has aimed to address the lack of emotional alignment capabilities in robots by developing a computational model of emotional alignment between older adults end-users and social robots. This model quantifies the congruency between a user’s identity and their perception of a social robot’s identity (19). We found that participants were more likely to use and enjoy pet-like social robots if there was congruency between their self-reported emotional identity and how they perceived a social robot. Emotional alignment between user and robot may lead to better end-user experiences with these devices. In this work, we aim to further uncover perspectives that users have around emotional alignment with robotic devices, including what emotional range from a social robot would be most ideal and whether there are limitations when it comes to robots displaying negative emotions.

Another key barrier to social robot adoption is the perception of stigma around assistive technology use by older adults (12, 33). Older adults have reported feeling embarrassed when interacting with the pet-like social robot PARO in front of others (12) and some may avoid bringing a humanoid social robot out in public with them (34). This stigma exists for several reasons; for example, assistive technologies meant to support ageing in place are often regarded as reflections of negative stereotypes of ageing, such as isolation and dependency (35, 36). Social robot design may also contribute to perceptions of stigma; although the studies cited above demonstrate perceptions of stigma around pet-like and humanoid social robots, unfamiliar, toy-like robots are also perceived by some older adults as childish and infantilizing (12, 21, 37). In order to increase acceptance and adoption of all types of social robots by older adults, it is essential to destigmatize the image of social robots. As a first step to addressing this barrier, it is necessary to consider the perspectives of end-users to identify gaps and areas for improvement in future social robot design.

The purpose of the present study was to explore ways of addressing the barriers to companion social robot adoption described above (lack of emotional alignment, stigma) through co-creation workshops with end-users. Using a combination of qualitative and quantitative methods, we report ideas for the design of an emotional social robot and considerations around public use of these devices from the perspectives of older adults, people living with dementia and care partners. This study had three research aims. Our first research aim was to replicate and extend other work on social robot design features and application areas (19, 21, 38) with an emphasis on social uses. The purpose of this aim was to facilitate comparability across studies and provide context for responses relating to the subsequent research aims. Our second research aim was to explore robot emotionality from the perspectives of end-users; specifically, we were in interested in whether participants would feel comfortable expressing their thoughts and feelings to a social robot, how wide an emotional range a social robot should display, and how it should display emotion. Finally, our third research aim was to learn key considerations around showing a robot to others in a home and public context, and whether stigma was a particular concern. By revealing end-user perspectives on emotional alignment and stigma, novel and tangible solutions can begin to be created for these two barriers to social robot adoption. Companion social robot devices designed with end-user feedback in mind will result in high-quality, user-centred social robots that are aligned with the stated priorities of older adults, persons living with dementia, and their care partners.

Our work is guided by the sociotechnical perspective, which regards technology use as dependent not simply upon a device’s design features, but upon human values – such as those of end-users and technology developers (39, 40). This perspective suggests that in order for a piece of technology to be truly successful in society, relevant societal factors must be understood and considered during development (40). In order to increase the potential for social robot adoption, we must gain a better understanding of the collective considerations around using these devices. Therefore, in this work, we engaged end-users in rich discussions about the nature of forming an emotional connection with a robot and the values associated with usage in larger social contexts with other people.

## 2. Materials and methods

This work was conducted in accordance with the Declaration of Helsinki and was approved by the University of British Columbia Behavioural Research Ethics Board (approval number H20-00762). We aimed to capture the opinions of older adults, people living with dementia, and care partners around social robot uses, emotion functionalities, and the potential for stigma around social robot use, so that future devices are better suited to their needs and priorities. This study took place in Vancouver, BC, Canada. We conducted online co-creation workshops, and we used a mix of qualitative and quantitative methods as seen in previous research in this field (21, 24, 35, 41). Please see Supplementary Figure 1 for a visual depiction of the study methods.

### 2.1. Input from lived experience expert group

Studies with older adults and persons with dementia conducted by our research group are completed in consultation with an advisory group of individuals with lived experience (the “Lived Experience Expert Group” or The League). We provide regular email updates to this panel on older-adult related projects. Four members of this panel provided feedback based on early drafts of the workshop agenda and participant recruitment materials, and they also participated in a pilot workshop to ensure that the workshops were sensitively constructed, used appropriately paced activities, and were generally well suited to older adults with and without dementia and care partners. The panel was updated on the preliminary findings of this project in a Zoom meeting, and they had a chance to comment on the results and provide ideas for the next steps of this work.

### 2.2. Participant characteristics

To recruit participants for the co-creation workshops, we used the following strategies: (1) social media postings through affiliated organisations (including Neuroethics Canada), (2) posters in community locations, (3) outreach through study partner organisations via email, (4) postings on the REACH BC Platform, (5) postings on the Neuroscience, Engagement and Smart Tech lab website, and (6) emails to individuals who had participated in prior research with our group who were interested in being re-contacted.

Our inclusion criteria for participation were (1) one of: older adults without a diagnosis of dementia (age 50+), older adults diagnosed with dementia (age 50+), or care partners for someone living with dementia (any age), (2) English fluency, (3) physical ability to participate in an online workshop, and (4) available for a 1.5-h online workshop. In our previous survey study on this topic (19), we included a wide age range for older adults, and based on the high number of respondents who self-identified between the ages of 50–59, we decided to make 50 our minimum age of inclusion for older adults in this study. Participants were asked to self-exclude if they had advanced cognitive impairment or a fear of robots or pets. Workshop participants were compensated with $50 gift cards to online retailers. Our sample size was finalised once we reached data saturation (please see “2.5 Analysis” section).

### 2.3. Pre-workshop consent and survey

Interested participants connected with a research team member via email. A follow-up phone call took place prior to the workshop to go over the study purpose, workshop logistics, and consent details. Verbal consent was obtained and recorded with permission for each participant. During this call, they were given the opportunity to become familiar with the video conferencing platform for the workshops (Zoom) if required. They were also invited to fill out an online pre-workshop survey before the date of the workshop with demographic information (gender and age), prior experiences with social robots, and the Multi-Dimensional Robot Attitudes Scale (MDRAS), which was intended to capture participants’ baseline attitudes toward social robots before the workshops (42). We evaluated five out of twelve constructs from this scale that were most relevant to our research questions and least reliant on prior robot experience: familiarity, interest, negative attitude, self-efficacy, and appearance.

### 2.4. Co-creation workshops

Using the Zoom online platform, we conducted seven 90-min workshops between July – August 2021. Four workshops were conducted with older adults, two were conducted with a mix of care partners and persons living with dementia, and one workshop was conducted with care partners only. Each participant attended only one workshop, and each workshop had 4–8 participants. Workshops were facilitated by one research team member (J.A.D.) with technical support from a research assistant (G.K.G.). The lead facilitator was a postdoctoral-level researcher with experience in engaging a wide range of technology end-users in qualitative research. The workshops began with an introduction to the research team and a short orientation to the Zoom platform. After participants introduced themselves and completed an icebreaker activity, we showed a 1-min and 16-s video of pet-like social robot MiRo (6) and the facilitator provided a short demonstration of MiRo via live video (∼1 min). Next, participants were invited to complete the Psychosocial Impact of Assistive Devices Scale (PIADS) (43) online based on their initial impressions of the robot. This scale captures the user’s perceived impact of assistive devices on their competence (items such as “independence,” “efficiency,” and “productivity”), adaptability (items such as “willingness to take chances,” “ability to participate,” and “ability to adapt to the activities of daily living”), and self-esteem (items such as “happiness,” “security,” and “self-confidence”).

Then, we showed participants a 2-min and 37-s video of a prototype socially assistive robot, T-Top, with a wide range of features and actions intended to show the breadth of possible applications for future social robots (44). These robots were meant to give all participants a similar baseline understanding of social robots. We chose MiRo and T-Top as exemplar social robots because they (1) are aesthetically distinct from one another, (2) represent different stages of development (e.g., prototype vs. commercially-available device), and (3) they showcase different functionality profiles that social robots can have. Please see Supplementary Table 6 for a summary of the differences between MiRo and T-Top.

The workshops then transitioned into a discussion period. The discussion period was not specific to MiRo and T-Top. There were three discussion topics to meet our three research aims: (1) social robot application areas and design elements, (2) emotion functionalities, and (3) the potential for stigma around social robot use. In order to replicate other work related to application areas and design elements, we asked open-ended questions about application areas and design, similar to previous works (19, 21, 38). Discussion questions within each topic were provided by the facilitator and follow-up questions were posed as needed. To facilitate the discussion, we used a mix of anonymous Zoom polls, live discussion, and prompts for participants to use the shared Chat function of the platform. The Zoom audio, chat, and poll results were recorded for analysis. Please see Supplementary Data Sheet 1 for the full workshop agenda, which was the same across all workshops and for all participants.

### 2.5. Analysis

#### 2.5.1. Quantitative analyses

**FIGURE 1:**
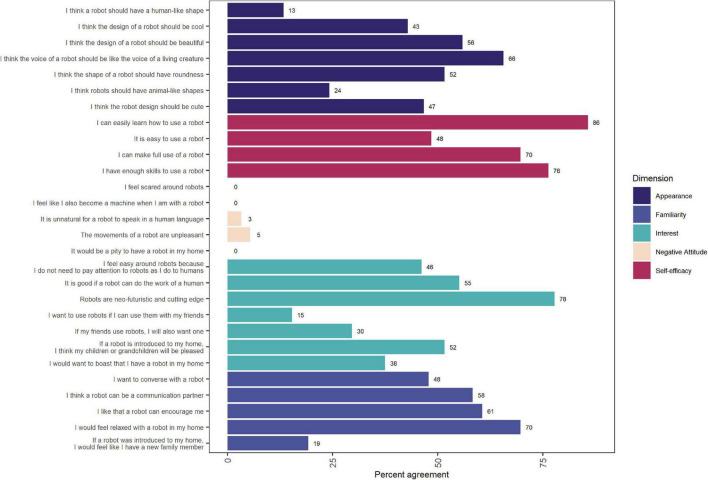
Agreement with items from the Multi-Dimensional Robot Attitudes Scale, delivered prior to workshop participation.

To capture participants’ attitudes toward robots before they attended our workshops, we circulated the MDRAS (42) to all participants before the workshops took place. For this instrument, respondents are asked to rank their agreement to statements that fall into each of these constructs, and responses are ranked on a seven-point Likert scale from −3 (not at all) to +3 (completely). Responses for each item across all participants were just % agreement as shown in Figure 1 reported as overall percentage agreement. To understand how participants felt a social robot would affect their well-being, autonomy, and quality of life, we had participants complete the PIADS (43) after we showed a video demonstration of MiRo during the workshops. For this tool, respondents are asked to rank the impact of a piece of assistive technology on each item, ranging from −3 (maximum negative impact) to +3 (maximum positive impact), with 0 indicating no impact. We calculated the mean and standard error of responses for competence, adaptability, and self-esteem separately for older adults, care partners, and older adults living with dementia. Finally, participants completed two multiple-choice Zoom polls during the workshop. The number of selections for each option were summed separately for older adult workshops and care partner and older adults living with dementia workshops.

#### 2.5.2. Qualitative analyses

The Zoom audio recordings and chat from the seven workshops were fully transcribed and analysed using conceptual content analysis, where the presence and frequency of concepts in texts are determined (45). We used an inductive coding approach (46) three researchers reviewed one transcript together to identify key themes, and an initial coding guide was developed. Two researchers then analysed another transcript independently using MAXQDA 2020 (47). Inter-rater reliability was determined by calculating the percentage of codes agreed upon over the total number of codes applied, and any ambiguities and potential changes to the coding guide were discussed. This process was repeated for subsequent transcripts until a final coding guide was developed and an inter-rater reliability of 85% was achieved, meaning that 85% of the total number of codes applied were applied the same way by both researchers. One researcher then coded all workshop data using the final coding guide. We opted to use a coding reliability approach, similar to other studies in this field (24, 30, 35) over a reflexive qualitative approach (48) in order to provide data on how often concepts were discussed, and thus provide guidance for which ideas should be prioritised in social robot development. We determined that data saturation was reached when the addition of workshops did not yield new emerging themes. See Supplementary Figure 1 for a visual depiction of the qualitative data analysis process and Supplementary Table 1 for the codebook used to code the data.

## 3. Results

### 3.1. Participants

A total of 44 participants attended the seven workshops: 25 older adults, 17 care partners, and two persons living with dementia. Older adults were 20 women and five men in their 50s to 80s, with the largest groups being 50s (*n* = 10) and 70s (*n* = 9). Seven (28%) had used a robot before. Care partners were 11 women and five men in their 30s to 70s, with the largest group in their 60s (*n* = 6). Seven (44%) had used a robot before. Demographic data was missing for one care partner. Both persons with dementia were men aged 65–74, one of whom had used a robot before. All but one participant (a care partner) indicated that they had owned a pet before.

### 3.2. Quantitative results

Results from the MDRAS and PIADS are reported here. When reporting this quantitative data, we also report a value that excludes the two participants with lived experience of dementia, labelled “ND.”

#### 3.2.1. MDRAS

We evaluated five constructs from the MDRAS (42): familiarity, interest, negative attitude, self-efficacy, and appearance (Figure 1). A majority of participants agreed with statements from the Self-Efficacy subscale, including “I can easily learn how to use a robot” (86%, ND: 85%) and “I have enough skills to use a robot” (76%, ND: 75%). Scores on the Negative Attitude subscale were low, reflecting a generally positive attitude toward robots, and no participant reported that they felt scared around robots. Scores on items around Interest were mixed, with 55% (ND: 52%) of respondents agreeing that robots should do human work and 52% (ND: 48%) reporting that their children or grandchildren would be happy if they introduced a robot into their home. Familiarity items were likewise mixed, with 58% (ND: 57%) of respondents saying that robots could be communication partners and 61% (ND: 65%) agreeing that robots could provide encouragement. In terms of the Appearance subscale, there was highest agreement with the idea that robots should have life-like voices (66%, ND: 67%) and lowest agreement with the statement that robots should have human-like shapes (13%, ND: 11%). These results are summarised in Supplementary Table 7.

#### 3.2.2. PIADS

**FIGURE 2:**
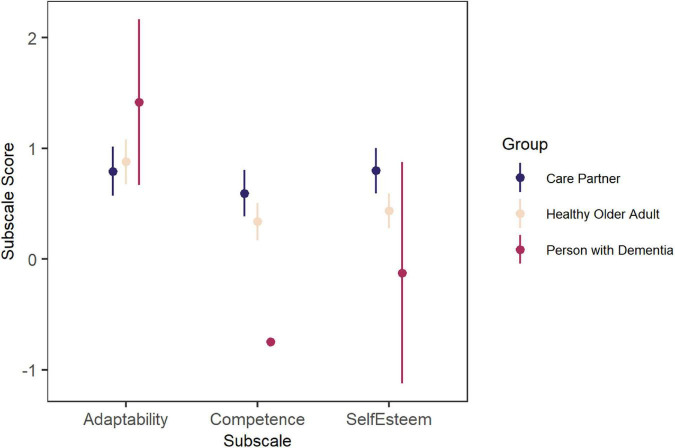
Responses to the PIADS – mean and standard errors are shown. Note that only two persons with dementia participated, so the error bars for this group are very large for adaptability and self-esteem and omitted for competence (for which only one participant responded).

The PIADS measures how the user believes assistive technologies will impact their competence, adaptability, and self-esteem. For each of these three subscales, a negative overall score indicates negative perceived impact, a score of zero indicates no perceived impact, and a positive overall score indicates positive perceived impact (43). On average, participants in all three groups (care partners, older adults, and persons with dementia) scored above zero for the adaptability subscale. Care partners and older adults scored above zero for the competence and self-esteem subscales. One person with dementia did not respond to the competence subscale questions, and the person with dementia who did respond scored below zero overall. For the self-esteem subscale, both persons with dementia had a mean score below zero (Figure 2). These results are summarised in Supplementary Table 8.

### 3.3. Qualitative results

Here, we discuss the major themes from analysis of the workshop data. All codes and their frequencies are listed in Supplementary Table 1. Quotes are identified as OA for older adults, CP for care partners, and PLWD for people living with dementia, along with a numeric participant code. Quotes are also marked with a workshop number: workshops 1–4 were conducted with older adults, workshops 5 and 6 were conducted with a mix of care partners and people living with dementia, and workshop 7 was conducted with care partners.

Within the broad theme of users, features, and applications, we discuss the following subthemes: (1) user groups that participants expressed would benefit from a social robot, (2) desired features for a social robot, and (3) desired non-social uses for a social robot. The social, emotional, and stigma-related considerations theme encompasses the following subthemes: (1) desired social uses for a social robot, (2) desired emotion recognition and response functionalities for a social robot, (3) the potential emotional impact of a social robot on the user, and (4) considerations around using a social robot around other people (including further subthemes user-centred considerations, robot-centred considerations, and audience-centred considerations). Finally, we report on the theme of social robot limitations shared by participants.

#### 3.3.1. Users, features, and applications

The first section of the workshops captured information about users, features, and applications as a way of introducing the topic and imagine application areas. Since these subjects are extensively covered in other works (19–21, 38), we will only briefly describe these findings.

##### 3.3.1.1. Users

In their discussions, participants mentioned various user groups that might benefit from social robots. These groups included older adults, people living with dementia, care partners, people who work from home, people who cannot have a pet, people who are lonely, and more. Several participants explained that isolation due to the COVID-19 pandemic has increased their desire for a social robot companion. See Supplementary Table 2 for all user groups discussed by participants and example participant quotes.

**FIGURE 3:**
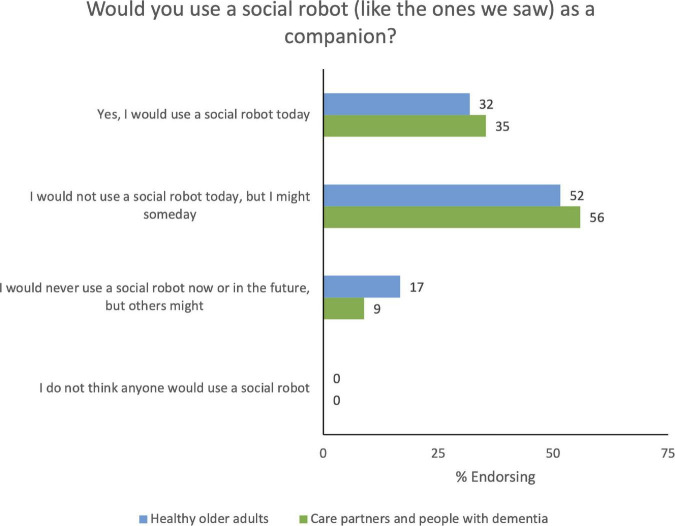
Poll: “Would you use a social robot as a companion?”

Participants also completed a poll and discussed whether they would currently use a social robot as a companion (see Figure 3 and Supplementary Table 9 for poll results). Most participants reported that they would not use a robot immediately, but they were open to it in the future; however, one participant claimed that they would use a robot right away: “I would definitely use the robot, referring to the video I saw, they can cook, they can serve for you, and they can also dance as well, and they are a super tool for human interaction. I would, I would take one in a minute” (Workshop 5, Participant CP-201). On the other hand, one participant said: “I hate to be the fly in the ointment, but I really just, I can’t see myself using one of those at all” (Workshop 1, Participant OA-306).

##### 3.3.1.2. Features

When discussing desired features of a social robot, a common topic raised was physical characteristics, with most participants expressing desire for a cute-looking robot with a furry texture. Another topic raised was detection and response capabilities; many participants wanted a robot that could detect their voice and produce speech. Finally, participants suggested that a robot could store data and connect to other devices, although privacy concerns surrounding data leakage and hacking were also highlighted. See Supplementary Table 3 for all features discussed by participants and illustrative quotes.

##### 3.3.1.3. Uses

In this section, we summarise social robot uses suggested by participants, excluding social uses which are discussed in Section “3.3.2.1 Social uses.” Participants desired a robot that could aid with housework, provide reminders, and be a source of entertainment. Participants also suggested functionalities to support health, such as monitoring symptoms of illness and calling for help in medical emergencies. See Supplementary Table 4 for all practical and health uses discussed by participants.

#### 3.3.2. Social, emotional, and stigma-related considerations

In this section, we describe the following topics in more detail: (1) social uses, (2) emotionality (emotion detection and production), (3) a robot’s impact on the user, and (4) considerations around using a robot around others.

##### 3.3.2.1. Social uses

With regards to social uses, participants discussed the potential for a robot to engage in conversation, non-verbal interaction, and functionalities to support connection between people. In general, some form of companionship, conversation or interaction with the robot was desired by most participants (“*Conversation, companionship, and interaction*” – 7/7 workshops), with one participant describing the robot as a “substitute friend” (Workshop 5, Participant PLWD-102). The importance of interaction with the robot is illustrated by a quote from one participant: “Being a cat person, a cat robot could purr. I feel that what is needed for most people, without or with dementia is being in relationship and having some form of communication.” (Workshop 7, Participant CP-218).

How such communication could take place was a common topic of discussion. For some, feelings of companionship and connectedness with a robot could occur with a one-way interaction, for example by the user “just saying good morning to [the robot]” (Workshop 2, Participant OA-312). For others, a two-way interaction was preferred; one participant described a robot that could carry “at least a limited conversation or at least respond to me as a pet would” (Workshop 1, Participant OA-307), and another participant said they wanted “a robot that felt like you were talking to another person as opposed to a robot” (Workshop 1, Participant OA-310). Some participants expressed that having a non-verbal reaction from a robot to indicate its attention was appropriate: “Even if it is just a little dog like one wagging its tail and shaking its head…if I was living alone, it would be nice to have something that I could just babble away to, and it would at least pretend to be interested in what I had to say” (Workshop 1, Participant OA-305).

Finally, participants illustrated the suitability of a robot as a medium for connecting with others (“*Supports connection between people*” – 7/7 workshops). One way a robot could achieve this purpose is by facilitating electronic communication between people; a robot could “use a phone” (Workshop 7, Participant CP-215) or be used for “answering emails, both reading them to me and writing the words down” (Workshop 7, Participant CP-216). One participant described how a robot could have telepresence applications: “So, one thing you could do is rather than having a facetime call with your phone, you could be looking at this robot whose head is a screen, and that would have an image of whoever you are talking to” (Workshop 1, Participant OA-310). Another way that a robot could support connection between people is by helping older adults living with dementia recognise those around them: “I could see, like say for instance somebody with Alzheimer’s, that face recognition and being able to say that name, so for somebody who is having trouble remember[ing] who people were” (Workshop 2, Participant OA-311). Likewise, another participant explained that “It would be great if the robot could show family pictures” (Workshop 7, Participant CP-217) to help people living with dementia remember their family members. A robot could also store narratives and stories to share with others, as illustrated by the following quote:

“I was thinking of it as a way to keep memories alive…my father, for example…he will tell stories from back when he was a kid…when he was going through school and university…gradually he will lose some of those memories…if it is recorded…it can be passed on to my kids so that they know about their grandfather, and it would be a little robot that gets passed through the generations with a bit of a history and story about my dad instead of doing it in writing” (Workshop 6, Participant CP-206).

Finally, the robot could contain storage for people living with dementia to “share thoughts” (Workshop 7, Participant CP-215) and “leave messages” (Workshop 7, Participant CP-215) for their care partner when they came home from work. In the words of one care partner: “At the end of the day when I would come home, to replay them together, not to violate his sense of privacy, but to be able to get into his, his life in real time for him so that we could then address issues that would improve his quality of life” (Workshop 7, Participant CP-215).

##### 3.3.2.2. Recognising and responding to emotions

One of the main topics of the workshops was robots and emotions. Participants commented on emotion recognition capabilities and potential emotional responses from the robot. One participant suggested that the user’s emotion could be detected through “the tone in our voice, or certain words it picks up” (Workshop 2, Participant OA-318).

**FIGURE 4:**
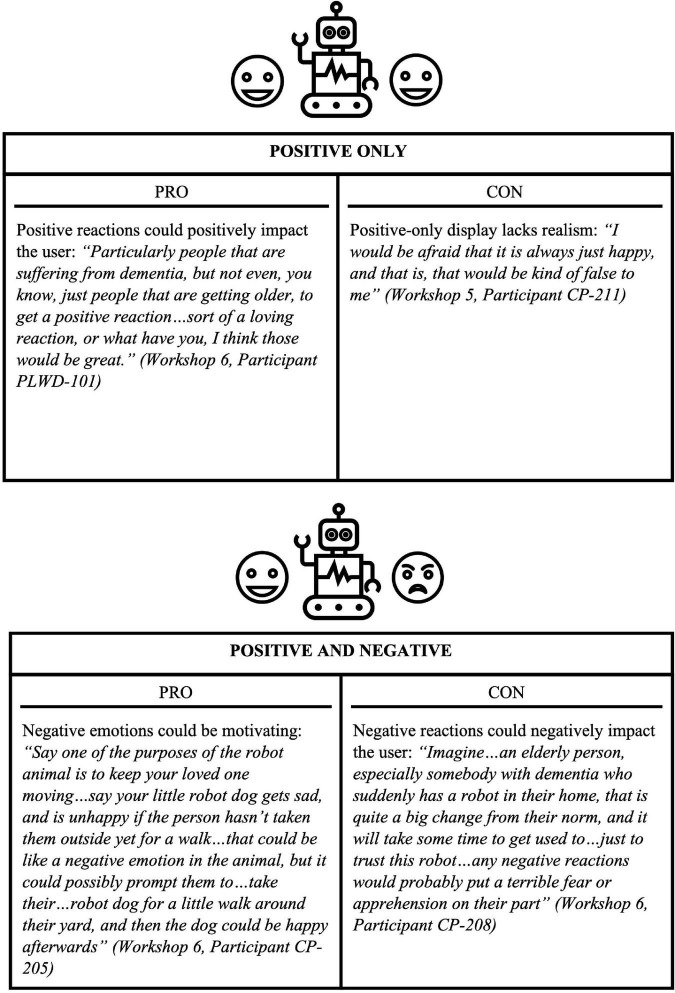
Pros and cons for social robot emotional ranges.

With regards to social robot displays of emotion, participants discussed whether a robot should display emotions, which emotions it should display, and what actions it should engage in to display such emotions. In general, most participants desired a robot with emotional display: “I think emotion for a social robot is almost mandatory, literally” (Workshop 6, Participant PLWD-101). One participant stated that they would want to control when the robot displays emotions: “I would like it to react, but I wouldn’t like it to be independent in its emoting, like if I give it…a voice command, or if I touch it, or if I do something first, go ahead, but I don’t want that thing running around and coming and putting its paw on me without being asked” (Workshop 4, Participant OA-320). As suggested by one older adult, a social robot could have a dial functionality to set interactivity: “It should have…low, medium, high interactive settings that you can change” (Workshop 2, Participant OA-314). When it came to the range of emotions a social robot should display, participants had mixed opinions. Some desired a robot with a wide range of emotions: “It is not just happy/sad, you know, delighted, distressed, I don’t think those are the two, you know, the only emotions that robots can exhibit” (Workshop 5, Participant CP-313). Others wanted more of a limited emotional display: “It would need to have some emotion, but I would like to keep it on the positive and the happy” (Workshop 6, Participant CP-203). See Figure 4 for a summary of pros and cons for different ranges of emotional display. Methods to express emotions suggested by participants included “facial expressions” (Workshop 1, Participant OA-310), “tail wagging” (Workshop 5, Participant CP-313), “ears flopping” (Workshop 6, Participant CP-208), and “cooing, or a positive noise” (Workshop 5, Participant PLWD-102).

Another topic discussed by participants was the capability for a robot to align its own emotions with the user’s emotions, or ‘emotional alignment’ (“*Produces emotionally aligned responses*” – 5/7 workshops). In general, participants agreed that emotional alignment was essential for a social robot companion, with one participant stating that “it has to meet you where you are at, your energy level, your emotion” (Workshop 2, Participant OA-311). Another participant explained how emotional alignment can lead to feelings of connection: “I mentioned that it should affirm the emotion of the person…and reinforce that emotion because that tells the person with dementia that [they are] connecting to something” (Workshop 5, Participant PLWD-102). To further illustrate the impact of emotional alignment, one participant described the downside of a robot without emotional alignment capabilities: “There is nothing more irritating than [being] overly cheerful when you are not in the mood” (Workshop 2, Participant OA-311). Likewise, the importance of accurate emotional alignment is demonstrated by the following quote: “I guess mirroring an emotion back to me, I guess it is responding in a way that it is assuming is mirroring, whether or not it is accurate is another question. I can see that creating frustration” (Workshop 3, Participant OA-317).

##### 3.3.2.3. Impacts on the user

**FIGURE 5:**
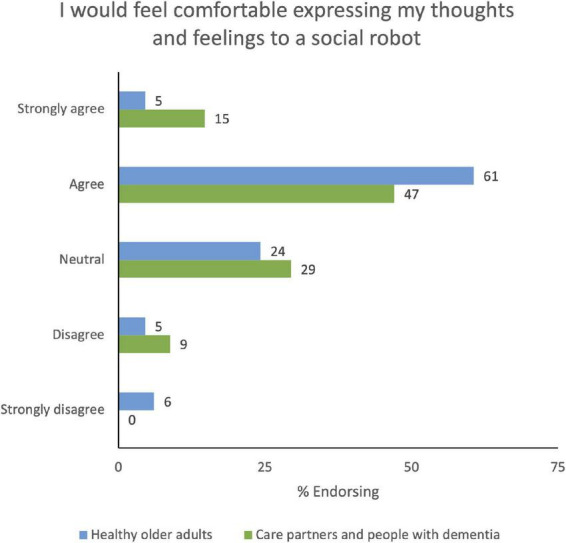
Poll: “I would feel comfortable expressing my thoughts and feelings to a social robot.”

Discussions from several participants reflected thoughts and concerns about the effect a robot may have on their well-being. To discover whether expressing thoughts and feelings to a robot would be comfortable for end-users, we had participants fill out a poll (Figure 5 and Supplementary Table 10).

The majority of participants agreed with the statement “I would feel comfortable expressing my thoughts and feelings to a social robot.” One participant explained that talking with a robot would be more comfortable than speaking to a real person: “I can say that I am not one to do excessive, or show my emotions, or talk about my emotions, so I am thinking that with a robot, because it is not a real person, that I would feel more comfortable” (Workshop 2, Participant OA-318). On the other hand, one participant did not see a purpose in expressing their thoughts and feelings to a robot: “I guess I see the existing examples as more of toys than as anything approaching the sort of companion, I would expect in terms of sharing my thoughts and feelings. It is something I do in confidence with people close to me…I guess I am not really uncomfortable; it is just that I wouldn’t see it as being fruitful” (Workshop 3, Participant OA-317). Privacy was also a consideration raised by several participants: “If I believe that the robot would not divulge whatever I have said, then I would feel comfortable” (Workshop 5, Participant PLWD-102).

On many occasions, participants described how being with a robot could improve their mood (“*Produces positive emotions or reduces negative emotions*” – 7/7 workshops), for example by alleviating feelings of loneliness: “I think it would just sort of alleviate that aloneness you sometimes feel, especially this last year with COVID” (Workshop 1, Participant OA-301). In terms of specific functions, one participant stated that they would like general “comfort functions that the robot is able to provide the patient” (Workshop 7, Participant CP-213) and another desired a robot that would “laugh with me” (Workshop 4, Participant OA-329). Finally, one care partner explained how having a robot monitor the person they care for could alleviate panic: “This would allow me to stop having those kind-of panicky moments and just be able to make sure everything was okay” (Workshop 7, Participant CP-213).

The potential for a robot to elicit negative emotions was also discussed (“*Produces negative emotions or reduces positive emotions*” – 6/7 workshops). For example, having a robot that constantly tries to cheer the user up in challenging times may make the user feel worse: “Without it, like again, trying to force you, that you feel more depressed because you are not following that” (Workshop 2, Participant OA-318). One participant explained the negative emotional experience that could arise with humanoid robots: “I think there are some ethical issues around deception. I wouldn’t want my mother to be intimidated or think she was talking with a human and then not to get the response she was expecting” (Workshop 5, Participant CP-313). Other negative emotional experiences are highlighted in other sections, including those that arise with inaccurate emotional alignment and negative emotional displays from a robot.

Another impact discussed was how a robot may affect the user’s autonomy (“*Affects autonomy*” – 4/7 workshops). This was a concern among several participants. For example, one participant mentioned the potential for a robot to influence how they view the world: “As soon as a robot starts affecting my emotions, then it is affecting my outlook on life, and I don’t know if I would be protected then…if things got out of control” (Workshop 2, Participant OA-303). Another participant expressed concern about a robot deciding what is best for them: “It could decide it knows better than you do what is good for you, and I wouldn’t like to have that” (Workshop 2, Participant OA-312). Several participants claimed that they did not want the robot to judge their capacity: “I wouldn’t want on the record my conversations with this robot used as a tool to assess my capacity, unless I was in really bad shape, if I couldn’t, you know, give consent or anything” (Workshop 2, Participant OA-314). In one of the workshops, a participant expressed concern about unwanted monitoring from the robot: “I feel I am suicidal, and it feels good to express that, not necessarily that I am going to kill myself…but then…somebody would intervene and call suicide prevention…then I would stop using the robot” (Workshop 5, Participant PLWD-102).

Other topics raised include the potential for a robot to improve clarity of thought and the ability to make decisions (“*Improves clarity of thought*” – 1/7 workshops), religious and cultural concerns about the concept of a robot such as “religious objections to humanoid computers” (Workshop 5, Participant CP-313) and the need for culturally-aligned physical design and speech recognition capabilities (“*Raises religious and cultural topics*” – 4/7 workshops), and ethical issues around deception and confusion (“*Causes confusion or deceives* – 3/7 workshops).

##### 3.3.2.4. Robots and others

**FIGURE 6:**
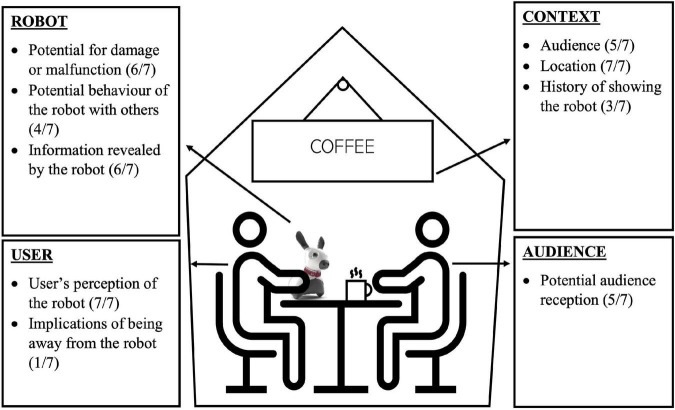
Considerations around showing a robot to other people. Codes are shown with bullet points, with frequencies in brackets.

The final major topic of discussion in the workshops was whether participants would use a social robot around other people. Participants were asked whether they would bring out their robot in front of other people in their home and in a coffee shop. Some participants were clearly comfortable with showing their robot to others: “Yes, I am comfortable showing it in my house or bringing it along to show off to my friends” (Workshop 5, Participant PLWD-102). Others were clear about not wanting to show a robot to others: “I will not bring MiRo” (Workshop 1, Participant OA-304). Considerations around showing a robot to others are grouped into user-, robot-, and audience-centred considerations in the following sections. See Figure 6 for a diagram summarising these considerations.

###### 3.3.2.4.1. User-centred considerations

Several considerations that were raised around showing a robot to others had to do with the user’s perception of the robot (“*User’s perception of the robot*” – 7/7 workshops). Many responses from participants illustrate a positive perception of social robots and their functionalities. For example, participants said they would show a robot to others to “demonstrate its capabilities” (Workshop 1, Participant OA-310): “I would like to show him off and all the things he/she/it can do” (Workshop 2, Participant OA-318). Another participant said they would show a robot to others due to “the opportunity to showcase…what it can do and how it has help[ed] me” (Workshop 3, Participant OA-321). For others, a robot would be considered “a part of my life” (Workshop 3, Participant OA-321), so they expressed a desire to bring a robot everywhere with them. On the other hand, some participants claimed that they would need to be “more comfortable at having it around” (Workshop 2, Participant OA-318) or “interested in it myself” (Workshop 3, Participant OA-317) before showing it to others. Finally, one participant stated: “At the present I would not show it as I do not think it is sophisticated enough” (Workshop 3, Participant OA-324).

Some participants viewed social robots as social devices, and therefore not useful or needed in a social context where interaction with other people was possible: “I am already socialising with my friends, so the social companionship of MiRo isn’t needed” (Workshop 6, Participant CP-205). One participant described a social robot as “a social thing, you know, at home” (Workshop 6, Participant CP-207). Another participant said they would not bring a robot out in public because they “want to be interacting with the people present” (Workshop 1, Participant OA-306).

Another user-centred consideration raised was the implications of being away from a robot (“*Implications of being away from the robot*” – 1/7 workshops). For example, one participant illustrated how a user could become emotionally attached to their robot, which would make leaving the robot at home difficult: “If somebody has become attached to this like little creature and…it is their friend, and it provides some companionship, then maybe they would be anxious about leaving it home alone potentially, and so bringing it might encourage them to go out” (Workshop 6, Participant CP-204). One participant explained that for people living with dementia, “[a robot] may…be helpful to keep the person calm” (Workshop 6, Participant PLWD-101) in public.

###### 3.3.2.4.2. Robot-centred considerations

With regards to robot-centred considerations, participants discussed the potential for damage to the robot or maintenance concerns (“*Potential for damage, malfunction, too heavy*” – 6/7 workshops), potential behaviour of the robot with others (“*Potential behaviour of the robot with others*” – 4/7 workshops), and the potential for private information to be revealed by the robot (“*Information revealed by robot*” – 6/7 workshops). With regards to potential behaviour of the robot, some participants felt that a robot could participate in social settings: “[The robot] could join in the conversation, and we would have a good old time” (Workshop 5, Participant CP-201). On the topic of information revealed by the robot, one participant said: “If it knew quite a bit of personal stuff about you, you wouldn’t want it blabbing out of control if you had visitors over” (Workshop 5, Participant OA-310).

###### 3.3.2.4.3. Audience-centred considerations

Many considerations had to do with the people that would see the robot (“*Potential audience reception*” – 5/7 workshops). Several participants said that they would show a robot to others if they felt that people would have a positive perception of their device. When asked if they would bring a robot out with other people present, one participant said: “I would bring Miro out. I think my friends would find it neat and want to see what the robot can do” (Workshop 1, Participant OA-301). Another consideration discussed was the potential “to get feedback about the idea of social robots” (Workshop 2, Participant OA-314) from others. Participants also commented on the opportunity to share the benefits of social robot technologies with others: “Sharing knowledge and new inventions is important” (Workshop 5, Participant CP-211). For example, one participant said they would “show off what it can be doing for your friends so that they know what is available out there for themselves or family members” (Workshop 2, Participant OA-318).

Concerns around judgement and stigma were also raised. One participant summarises this issue upon answering the question of bringing a robot out in public: “Would I be open to that, yes. A lot of people wouldn’t be though because stigma, discrimination, being stared at, questions, you know, misconceptions about oh, there something is something really wrong with them, so I think that there needs to be some sort of a balance” (Workshop 7, Participant CP-218). On one hand, one participant felt that a social robot could be a socially acceptable device to have in public: “I think it is acceptable to have…an electronic gadget, like that is how it could be perceived by the public, as an electronic gadget…It doesn’t need to be associated with illness” (Workshop 5, Participant CP-313). On the other hand, another participant illustrated the potential for stereotyping and describes one way she could be negatively portrayed by the public: “There is that eccentric lady bringing her little robot with her to the coffeeshop again” (Workshop 6, Participant CP-206). Several participants highlighted the potential for attracting negative attention and judgement from others. One care partner stated: “If people didn’t know that I was having some kind of deficiency, or like that I have been diagnosed with dementia, it might raise questions to other people” (Workshop 5, Participant CP-211). Another participant also shared their concerns: “As far as taking it outside of the home, I have grave concern that people would, first of all, question my competence in whatever I do” (Workshop 7, Participant CP-216).

In contrast, several participants described how a social robot may actually help to fight negative stereotypes around ageing, dementia, and different types of disabilities. In the words of one participant: “I would take it wherever I am that would spark interest, and it would I think open the eyes of other individuals. I don’t want to be stereotyped. You can look at me and you don’t know there is anything wrong with me…this would open up people’s minds, I think, to more acceptance of individuals who have special needs” (Workshop 7, Participant CP-218). One participant living with dementia explained that showing a robot to others could help “them to understand that I am able to deal with something in my life by using a device” (Workshop 5, Participant PLWD-102). On a broader scale, participants suggested that a robot could help raise awareness of dementia, as illustrated by one example scenario: “being able to say yeah, this is our companion, and his name is, her name is whatever, and we have it because my husband lives with dementia, and this allows him to remain more functional, and just that ability to be in the public space, and use that as a calling card, it sounds to me like a terrific use” (Workshop 7, Participant CP-215).

#### 3.3.3. Limitations of social robots

Within all the workshop topics and discussions, there were various limitations of social robots that were raised. These are also summarised in Supplementary Table 5. The most frequent limitation discussed was practical use (6/7 workshops). Several participants felt that social robots are not advanced enough, and they often compared social robots to kids’ toys: “Social robots are really a toy. Mostly they would encourage your grandkids to come and visit” (Workshop 4, Participant OA-330). Some participants felt that current social robot functionalities have limited practical use in their day-to-day life: “I mean if it was going to vacuum my floor, or translate a foreign language for me, or have a useful purpose, but what I have seen so far is not something I would be really interested in” (Workshop 1, Participant OA-306). Some participants felt that social robots have limited dementia functionality (3/7 workshops): “And with Miro, I actually like Miro, but I would like to see a little bit more usefulness in terms of the tasks that it could do, especially for somebody living with dementia. I would like to see more interaction” (Workshop 6, Participant CP-208).

Another limitation discussed was limited social capacity (5/7 workshops). One participant explained that interaction with a robot does not quite replace human interaction: “I am not quite sure they will actually replace another person in terms of how you feel when you are dealing with them, and to me, that would be the objective, to have a robot that felt like you were talking to another person as opposed to a robot” (Workshop 1, Participant OA-310). One participant described limited voice recognition capability as a barrier to social interaction: “I am not particularly impressed with the voice recognition capability, and I think voice recognition capability is an essential requirement for a robot to try and act socially” (Workshop 1, Participant OA-310). Another participant mentioned that context-appropriate responses are difficult to programme: “in terms of knowing what the context is to give the appropriate response, I think that still is really, really very, very difficult” (Workshop 5, Participant CP-313). One participant summed up this topic by saying: “I can see it as being fun and interesting, but not necessarily deeply fulfilling in terms of what I expect from a companion” (Workshop 3, Participant OA-317).

Finally, participants raised the potential for damage or harm (6/7 workshops), either to the robot or to the user. Regarding the social robot T-Top shown as a demo in the workshops, one participant said they “didn’t like the wires, and those wires, it looks very fragile to me. It looks like if it toppled it would break very easily” (Workshop 6, Participant CP-208). Several participants explained how the robot could be a “safety and tripping hazard” (Workshop 1, Participant OA-306). One participant described this further: “You definitely want it to be fast enough to get out of your way if you were, you know, walking and, yeah, I mean tripping and falling is a huge concern of seniors, and so you wouldn’t want to trip over the thing” (Workshop 1, Participant OA-305).

## 4. Discussion

This study builds on prior work on social robotics for older adults and uncovers new critical considerations around emotionality and stigma. Through the lens of the sociotechnical perspective, social robots should not just be considered as physical pieces of technology with several functionalities, but also as devices that are shaped by social systems that can have varying impacts depending on end-user values and beliefs. Robot emotional capability is a key priority for researchers and technology developers (27, 28); however, before development, it is essential to consider end-user values around emotional range and alignment. Similarly, although societal stigma around ageing and assistive technologies has been recognised as a key barrier to device adoption (12, 33), few studies have explicitly explored end-user perspectives on stigma around social robots. Altogether, the findings from this work will support the development of user-centred, emotionally aligned social robots.

We conducted workshops with small groups of participants. By taking a qualitative approach, we were able to capture individual narratives to supplement the survey data in our previous related work on developing a computational model of emotional alignment for social robotics (19). Several other works have also aimed to incorporate older adult end-user perspectives into social robot development through end-user narratives (21, 49); however, these works have primarily focused on robots and populations in long-term care and nursing home contexts. In this work, we pictured location-agnostic robots for end-users with a wider range of housing experiences.

In our exploration of social robot design features and application areas, we considered opinions on all types of functionalities, rather than just companionship (50), by asking very open-ended questions, inspired by several other works (19, 21). Many of our findings on this topic align with previous research, such as the desire for a soft and furry pet-like robot (19, 21), the potential for a socially assistive robot to complete practical tasks such as housework (19), the need for social interactivity for companion social robots (21, 51), and general concerns around privacy (19, 24, 52). Since the above functionalities and applications overlap between research works, they should be made key priorities for future pet-like and assistive social robots.

On the topic of communication with a social robot, the majority of our participants desired some form of interaction with a companion social robot; however, limited social capability was highlighted by several participants as a barrier to meaningful communication with a robot. For example, one participant said interaction with a robot would not replace human interaction, and another explained that they saw social robots as toys rather than something to express thoughts and feelings to. Furthermore, our MDRAS results show mixed opinions on whether a robot could fulfil the role of a communication partner. In another study, a lack of language and emotional capabilities of robots was revealed as a barrier to communication with a social robot (52). While some of our participants similarly expressed desire for a companion social robot that would have human-like conversational and emotional abilities, several participants wanted a robot that simply produced non-verbal responses to the user to indicate it pays attention. Most participants did express desire for an expressive robot that produces emotionally appropriate and aligned responses to the user. With regards to how companion social robots should display emotion, we heard a wide range of suggestions; similar to findings from previous research (21), our participants suggested widely recognised displays of emotions, including pet-like actions such as wagging tails and positive noises. As expected, our results suggest that interactive ability and robust emotion functionalities are necessary features for high-quality social robots intended to serve as companions, such as pet-like devices.

In the workshops, we investigated the impact that a social robot might have on the user’s emotional well-being. According to our results from the MDRAS scale administered before the workshops, participants generally did not have negative attitudes toward social robots, and the poll results reflect general agreement that expressing thoughts and feelings to a social robot would not be an uncomfortable experience. Data from the PIADS instrument delivered during the workshops demonstrate that care partner and older adults feel a social robot could positively impact their emotional wellbeing; however, results from the people living with dementia who participated illustrate a perceived negative impact on their emotional wellbeing. In several workshops, the negative emotional impact that a robot could have on the user was discussed, for example due to inadequate or inaccurate emotional alignment or negative emotional display from a social robot. We document a range of opinions around whether a robot should display negative emotions, but there was general agreement that emotional alignment was a necessary feature for companion social robots, and we suggest that social robots intended to serve as companions (for example, pet-like devices) should have high-quality emotion detection and response functionalities that allow the device to accurately align its emotions with the user. Additionally, a few participants suggested that they would like to control a social robot’s emotions, and adaptable emotional display for a pet-like social robot depending on the user’s preferences has been reported as desirable in other studies (21). The different preferences for social robot emotional range expressed during workshops strongly support the development of companion social robots with a customizable level of emotion and interactivity.

To explore the topic of stigma, we asked participants whether they would want to show a robot to other people in their home or in a coffee shop. Participants highlighted several benefits to presenting a robot to others, including the opportunity to share the benefits of new technologies and raise awareness of ageing and dementia. On the other hand, they raised several downsides, including the potential for stigma, discrimination, and attracting negative attention from other people. In general, research suggests that older adults may feel stereotyped when using technology. For example, they may feel embarrassed or anxious if they experience difficulty while using technology (52, 53). Older adults may also recommend assistive technologies for people who experience isolation or disability (35). Through the lens of the sociotechnical perspective, these findings demonstrate an overall tension in the ‘social’ aspect of social robot technologies, between the positive and negative aspects of a robot being a shared social experience. To apply these findings to the creation of social robots, robot developers and researchers should carefully consider what side of this tension their end-users are likely to experience and design a social robot to operate accordingly. For example, the appearance of social robots should be carefully designed, as previous research has shown that pet-like, humanoid, and toy-like devices may elicit feelings of stigma (12, 21, 37). Social robot applications, such as the ability to converse, should also have complexity and nuance to avoid infantilization. There is a critical need to destigmatize social robot devices in order to facilitate acceptance, and our results demonstrate a path forward; rather than being viewed as reflections of negative stereotypes of ageing, social robots could be regarded as conversation starters and an opportunity to educate others about ageing, dementia, and how different types of technology can provide high-quality support for older adults with and without dementia and their care partners. These findings have implications not only for the design of social robots but also for the marketing and advertisement of devices; emphasising the potential for facilitating connections among people and educating others may be a way to destigmatize social robots.

Though there are strengths to the approach used in this work, there are also several limitations. Certain demographic information was not collected, including place of residence, ethnicity, living situation, or care partner relationships, which limits the generalizability of our findings. Although these pieces of information were not central variables for the questions under study, they should be explored in future work. We chose an inclusive age range of 50+ for older adults with and without dementia based on respondents in our previous survey study (19); however, people on the lower end of this age range are often still an active part of the workforce, and other studies on social robots typically have a higher minimum age for older adult inclusion (24, 50). Holding the focus groups over Zoom was necessary given the COVID-19 pandemic restrictions; however, since physical interaction with the social robots was not possible, feedback on tactile, visual, and auditory features was likely limited. Previous research has also shown that perceptions of social robots may change after interacting with them (54). As an alternative, we showed short video clips of two existing social robots and the facilitator carried out a demonstration on camera; however, there may not have been enough time or information provided about social robots for participants to fully form opinions and feedback about the devices during the workshops. Most of our recruitment avenues were online and the focus groups were held over Zoom, which suggests that participants in this study were likely experienced in using technology. As a result, they may have been more open to the idea of social robots than older adults who are not as experienced with technology. Furthermore, research shows that prior attitudes toward robots influences how people will evaluate them (55), so our results may have been skewed. Additionally, although the robots shown in the video demos, MiRo and T-Top, demonstrate various stages of social robot development and a wide range of features, these robots are not as widely studied as other devices, such as Paro (12, 21, 24, 56, 57), which limits comparability across studies. We chose not to restrict our workshop discussions to specific types of social robots, and although this allows for a wide range of imaginable application areas and design features for a social robot, it also limits nuanced discussion about the different types of social robots that currently exist, such as pet-like robots. As a result, some of our findings are more applicable to one type of social robot over another. Technology developers and researchers should consider implementing the results from this work that are most relevant to their device of specialty. Finally, we had only two participants living with dementia due to challenges with recruitment, which limits the potential for dementia-specific feedback, as well as in-depth comparisons of the perspectives between groups. Instead, we provide descriptive statistics to summarise our quantitative results, paint a picture of our sample, their experiences, and baseline attitudes, which provides a foundation for the interpretation of the qualitative data.

Despite its limitations, this work contributes to the advancement of social robot development for older adults, people living with dementia and care partners. We explored end-user opinions around social robot emotionality and the potential for stigma – two under-explored barriers to social robot adoption – which will ultimately contribute to the design of future devices that are more beneficial and emotionally appropriate for end-users. These results will be applied by technology developers, including the teams that developed social robots MiRo and T-Top showed during our co-creation workshops, to build on existing platforms to make them better suited for end-users. Future research should evaluate the quality of end-user experiences after adopting newly developed social robots to ensure that new devices are as impactful, meaningful, and beneficial for end-users as they are intended to be.

## Data availability statement

The raw data supporting the conclusions of this article will be made available by the authors, without undue reservation.

## Ethics statement

The studies involving human participants were reviewed and approved by The University of British Columbia Research Ethics Board. The patients/participants provided their written informed consent to participate in this study.

## Author contributions

JD and JR: conception and design of the work. JD and GG: participant recruitment and facilitating the co-creation workshops. JD, JK, and JR: data analysis and interpretation. JD and JK: writing the first draft of the manuscript. JR: supervision. All authors helped to revise the manuscript, approved the submitted version of the manuscript, and agreed to be personally accountable for their contributions.
