# Supplementary material for: Older adult perspectives on emotion and stigma in social robots
Source: Front Psychiatry. 2023 Jan 12;13:1051750. doi: 10.3389/fpsyt.2022.1051750 (PMC9878396; doi:10.3389/fpsyt.2022.1051750)
Supplement: Supplementary file 1 [file Data_Sheet_1.docx]

1. Welcome and introductions, including a summary of project goals, a Zoom tutorial, and a recap of study consent details.
2. Icebreaker activity
3. Robot demo I: MiRo video
4. Completion of the PIADS
5. Robot demo II: TTop video
6. Discussion topic 1: Uses

| **Mode of data collection** | **Question** | **Details** |
| --- | --- | --- |
| Zoom poll | “Would you use a social robot (like the ones we saw) as a companion?” | Participants completed the Zoom poll and were then asked to explain their answer choice. |
| Zoom audio or chat box | “Are there elements of the social robots’ design that would make you more or less interested in using them as companions?” | Participants were asked to answer this question out loud or with the Zoom chat box. |
| Zoom chat box | “If you were to use a social robot based on the ones we saw as a companion, what would you like it to do for you?” | Participants were asked to type their answers in the Zoom chat box, and the facilitator put the answers on a PowerPoint slide which was screenshared to the group. Participants were asked to discuss suggested applications further. |

1. Discussion topic 2: Emotions

| **Mode of data collection** | **Question** | **Details** |
| --- | --- | --- |
| Zoom poll | “I would feel comfortable expressing my thoughts and feelings to a social robot (based on the ones we saw)” | Participants completed the Zoom poll and were then asked to explain their answer choice. |
| Zoom audio or chat box | How much emotion should a social robot display back to the user?” | Participants were asked to answer this question out loud or with the Zoom chat box. |

1. Discussion topic 3: Stigma
   1. Scenario I: You’re having coffee with a few friends at your home. Would you bring MiRo out? Why or why not? Participants were asked to share their answers in the chat.
   2. Scenario II: You’re having coffee with a few friends at a coffee shop. Would you bring MiRo out? Why/why not? Participants were asked to share their answers in the chat.
   3. Participants were then asked to discuss their answers to both scenarios out loud.

**Data Sheet 1.** Facilitator’s guide for the workshops.
