# Supplementary figures and images for: Older adult perspectives on emotion and stigma in social robots
Source: Front Psychiatry. 2023 Jan 12;13:1051750. doi: 10.3389/fpsyt.2022.1051750 (PMC9878396; doi:10.3389/fpsyt.2022.1051750)

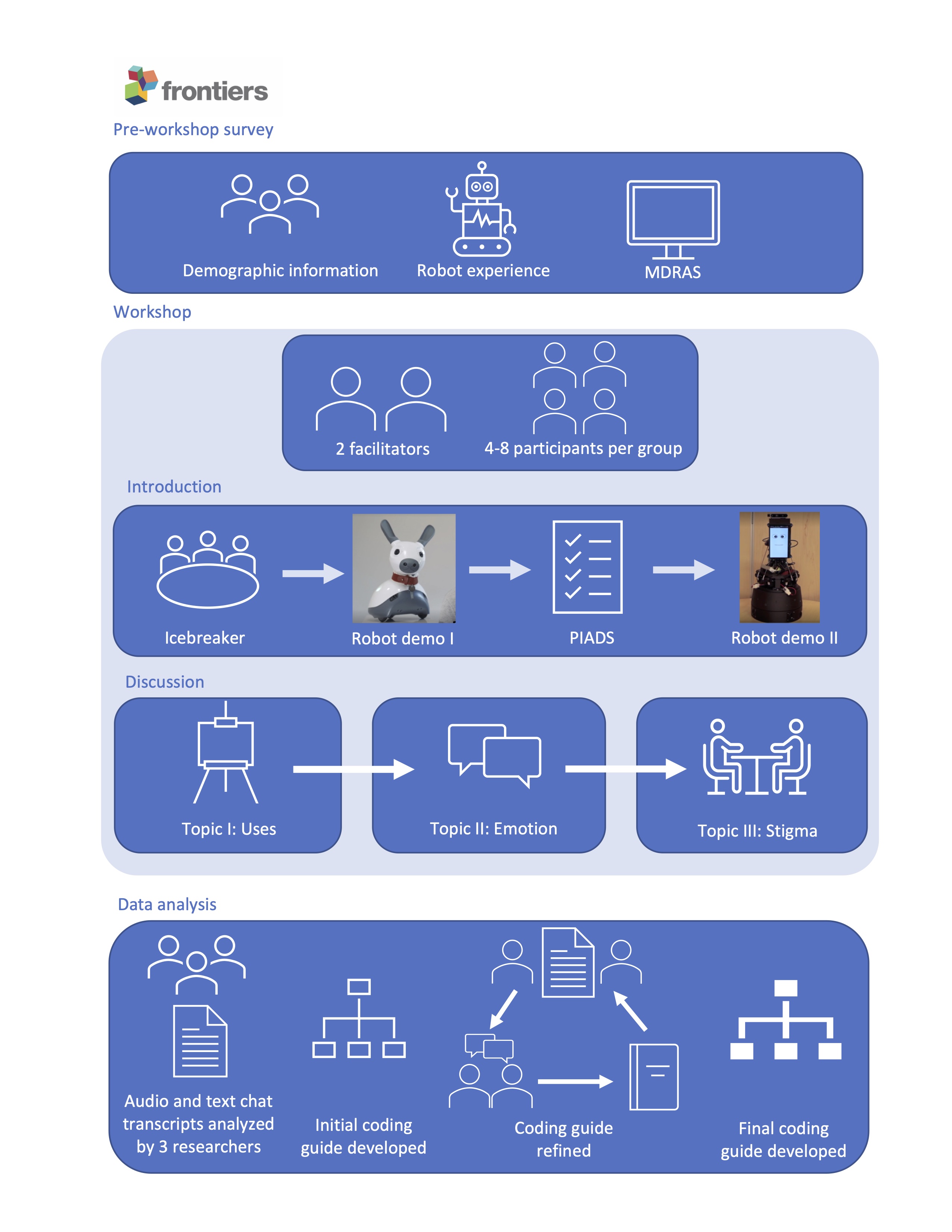

Supplement: Supplementary file 2 [file Image_1.JPEG]
