# Supplementary material for: Older adult perspectives on emotion and stigma in social robots
Source: Front Psychiatry. 2023 Jan 12;13:1051750. doi: 10.3389/fpsyt.2022.1051750 (PMC9878396; doi:10.3389/fpsyt.2022.1051750)
Supplement: Supplementary file 3 [file Table_1.DOCX]

**Table 1.** Code system and frequencies

| **Code System** | **Frequency** |
| --- | --- |
| Code System | 1384 |
| Uses and Features |  |
| Features and applications of a social robot |  |
| Data and programming features of the robot |  |
| Data storage, access, and sharing | 27 |
| Permissions and customization | 21 |
| Security and privacy | 27 |
| Detection and identification abilities of the robot |  |
| User's identity | 3 |
| User's voice | 27 |
| User's movement | 3 |
| User's emotion | 8 |
| User's touch | 4 |
| Events in the environment (not from user) | 14 |
| Potential responses of the robot |  |
| Visual display | 17 |
| Sounds and speech | 31 |
| Movement and locomotion | 48 |
| Facial expressions and displaying emotions | 30 |
| Produces emotionally aligned responses | 9 |
| Specific applications or tasks for the robot |  |
| Serve | 4 |
| Cooking, cleaning, housework | 19 |
| Conversation, companionship, and interaction | 116 |
| Provide information | 38 |
| Reminders, check-ins, suggestions | 49 |
| Facilitate entertainment | 11 |
| Supports connection between people | 26 |
| Treats illness, supports or monitors health | 47 |
| Physical safety | 31 |
| Repair and maintenance of the home | 4 |
| Physical touch | 22 |
| Physical appearance of the robot | 44 |
| Personality of the robot | 10 |
| Processing capacity | 22 |
| Maintenance considerations | 9 |
| Limitations of robots |  |
| Social capacity | 12 |
| Practical use | 17 |
| Dementia functionality | 4 |
| Potential for damage/harm | 15 |
| Comparators: items to which the social robot was compared |  |
| Electronics and objects | 60 |
| Toys | 13 |
| Person | 40 |
| Media character | 7 |
| Real animal | 41 |
| Users |  |
| My current or past need or desire for a social robot |  |
| Yes | 12 |
| No | 11 |
| My future or hypothetical need or desire for a social robot |  |
| Yes | 5 |
| No | 2 |
| User groups |  |
| Alone or lonely | 31 |
| Disability |  |
| People with dementia | 51 |
| Non-dementia disability | 24 |
| Pet status | 8 |
| Housing and employment | 13 |
| Older adults | 10 |
| Care partners | 5 |
| Children | 3 |
| Emotions, Thoughts, and Feelings |  |
| Impacts on user |  |
| Affects autonomy | 10 |
| Affects mood | 2 |
| Produces negative emotions or reduces positive emotions | 13 |
| Produces positive emotions or reduces negative emotions | 22 |
| Improves clarity of thought | 1 |
| Causes confusion (or deceives) | 11 |
| Raises religious and cultural topics | 7 |
| Robots and others |  |
| Do I want to show my robot to others? |  |
| Yes | 41 |
| No | 23 |
| Considerations around showing a robot |  |
| Implications of being away from the robot | 2 |
| Audience | 15 |
| Location | 20 |
| History of showing the robot | 4 |
| User's perception of robot | 34 |
| Potential audience reception | 34 |
| Potential for damage, malfunction, too heavy | 19 |
| Potential behaviour of the robot with others | 7 |
| Information revealed by robot | 14 |
