# Supplementary material for: Older adult perspectives on emotion and stigma in social robots
Source: Front Psychiatry. 2023 Jan 12;13:1051750. doi: 10.3389/fpsyt.2022.1051750 (PMC9878396; doi:10.3389/fpsyt.2022.1051750)
Supplement: Supplementary file 4 [file Table_2.DOCX]

**Table 2.** Social robot user groups suggested by participants.

| **Theme** | **Subtheme** | **Example** | **Frequency** |
| --- | --- | --- | --- |
| User groups | Older adults | “I’m thinking of a senior living alone who is maybe socially isolated, especially during covid” (Workshop 6, Participant CP-208) | 5/7 workshops |
|  | People living with dementia | “I think it is lovely to have that companionship, and I could see a real use for that…for somebody with dementia, especially somebody living alone, to interact and to express their thoughts, and their ideas” (Workshop 6, Participant CP-208) | 5/7 workshops |
|  | Care partners | “Now I am thinking about the caregiver’s point of view” (Workshop 4, Participant OA-325) | 3/7 workshops |
|  | People living with a physical disability or mental illness | “With people with depression…just to not feel completely alone when there is no one there” (Workshop 2, Participant OA-314) | 6/7 workshops |
|  | People who live alone or who are experiencing loneliness | “After a year of covid social isolation, I am probably leaning more towards any type of social interaction from a robot than I would have a year ago” (Workshop 3, Participant OA-319) | 7/7 workshops |
|  | Children | “Social robots are really a toy. Mostly they would encourage your grandkids to come and visit” (Workshop 4, Participant OA-330) | 2/7 workshops |
|  | People with a specific housing or employment situation | “So, I am still working, so I don’t see the need for myself to be accompanied...when you stay home by yourself, like my husband worked a year and a half in the past by himself at home, so I think that companionship is valuable” (Workshop 1, Participant OA-304) | 7/7 workshops |
|  | People who cannot have a pet | “I live alone, and I can’t have a pet where I live, and yeah, just something to talk to instead of just myself” (Workshop 1, Participant OA-301) | 4/7 workshops |
