# Supplementary material for: Older adult perspectives on emotion and stigma in social robots
Source: Front Psychiatry. 2023 Jan 12;13:1051750. doi: 10.3389/fpsyt.2022.1051750 (PMC9878396; doi:10.3389/fpsyt.2022.1051750)
Supplement: Supplementary file 5 [file Table_3.DOCX]

**Table 3.** Social robot features suggested by participants.

| **Theme** | **Subtheme** | **Example** | **Frequency** |
| --- | --- | --- | --- |
| Physical characteristics | N/A | “The cuter it is, the more likely you will engage with it” (Workshop 5, Participant CP-313) | 7/7 workshops |
| Detection capabilities | Identity recognition | “For instance, somebody with Alzheimer’s, that face recognition and being able to say that name, so for somebody who is having trouble remember[ing] who people were, that would be a big help for that person” (Workshop 2, Participant OA-311) | 3/7 workshops |
|  | Voice recognition | “The possibility for [a robot] to understand and react in other languages” (Workshop 6, Participant CP-205) | 6/7 workshops |
|  | Movement recognition | “The robot could…ha[ve] the capability of following somebody around by hearing them walking” (Workshop 1, Participant OA-310) | 1/7 workshops |
|  | Touch recognition | “Maybe it makes a sound, like it purrs when you pet it” (Workshop 4, Participant 311) | 2/7 workshops |
|  | Detecting the environment | “The smell of gas and ability to alert someone that the stove is on” (Workshop 7, Participant CP-215) | 5/7 workshops |
| Responses | Sounds and speech | “Even if not comprehensive conversation – having a voice in the background is important” (Workshop 7, Participant CP-215) | 7/7 workshops |
|  | Visual display | “Have it respond with lights” (Workshop 1, Participant OA-301) | 7/7 workshops |
|  | Movement | “Moving body parts e.g., ears, eyes, etc.” (Workshop 6, Participant CP-208) | 6/7 workshops |
| Data and programming | Data storage, access, and sharing | “Having it connected to our phones or computers would elevate this into something really practical as well as companionship” (Workshop 1, Participant OA-306) | 7/7 workshops |
|  | Permissions and customization | “I don’t want it being independent. I want it to be programmed to do the things I want it to do and in reaction to the things that I do” (Workshop 4, Participant OA-320) | 7/7 workshops |
|  | Security and privacy | “I’d have concerns about privacy due to the potential for hacking” (Workshop 3, Participant OA-322) | 7/7 workshops |
| Other features | Personality of the robot | “My robot would be very funny and have a very warped sense of humour” (Workshop 7, Participant CP-218) | 5/7 workshops |
|  | Processing capacity | “Yeah…it could do some processing, but most of the processing could be done in the powerful computer or phone you have, but the robot could display it in a much more natural human-type way that people would appreciate more” (Workshop 1, Participant OA-310) | 7/7 workshops |
|  | Maintenance considerations | “You get a fuzzy robot that you can’t wash, I imagine, so I mean that is just practical, they go grey” (Workshop 2, Participant OA-312) | 3/7 workshops |
