# Supplementary material for: Older adult perspectives on emotion and stigma in social robots
Source: Front Psychiatry. 2023 Jan 12;13:1051750. doi: 10.3389/fpsyt.2022.1051750 (PMC9878396; doi:10.3389/fpsyt.2022.1051750)
Supplement: Supplementary file 6 [file Table_4.DOCX]

**Table 4.** Practical and health uses for a social robot suggested by participants.

| **Theme** | **Subtheme** | **Example** | **Frequency** |
| --- | --- | --- | --- |
| Practical uses | Serve | “It could…get me a drink” (Workshop 7, Participant CP-219) | 4/7 workshops |
|  | Cooking, cleaning, housework | “I would like it to perform tasks around the house” (Workshop 6, Participant CP-208) | 7/7 workshops |
|  | Repair and maintenance of the home | “I wouldn’t mind it being able to repair problems with the computer” (Workshop 2, Participant OA-311) | 4/7 workshops |
|  | Provide information | “Respond to questions that I might ask regarding news and weather” (Workshop 6, Participant PLWD-101) | 7/7 workshops |
|  | Reminders, check-ins, suggestions | “I know I have missed lots of messages because, you know, my phone is accidentally turned off… it could even say…there is a message for you” (Workshop 1, Participant OA-301) | 7/7 workshops |
|  | Facilitate entertainment | “Read me ebooks” (Workshop 4, Participant OA-320) | 5/7 workshops |
| Health uses | Treats illness, supports or monitors health | “Maybe it might take my temperature and notices that I have a fever, or that my heart rate is too high, and then it…calls a doctor” (Workshop 4, Participant OA-311) | 7/7 workshops |
|  | Physical safety | “If I fell and couldn’t get up, would it be able to, you know, notify, I don’t know an emergency contact” (Workshop 3, Participant OA-319) | 7/7 workshops |
