# Supplementary material for: Older adult perspectives on emotion and stigma in social robots
Source: Front Psychiatry. 2023 Jan 12;13:1051750. doi: 10.3389/fpsyt.2022.1051750 (PMC9878396; doi:10.3389/fpsyt.2022.1051750)
Supplement: Supplementary file 7 [file Table_5.DOCX]

**Table 5.** Limitations of social robots.

| **Theme** | **Example** | **Frequency** |
| --- | --- | --- |
| Practical use | “I mean if it was going to vacuum my floor, or translate a foreign language for me, or have a useful purpose, but what I have seen so far is not something I would be really interested in” (Workshop 1, Participant OA-306) | 6/7 workshops |
| Limited dementia functionality | “And with Miro, I actually like Miro, but I would like to see a little bit more usefulness in terms of the tasks that it could do, especially for somebody living with dementia...I would like to see more interaction” (Workshop 6, Participant CP-208) | 3/7 workshops |
| Social capacity | “I am not quite sure they will actually replace another person in terms of how you feel when you are dealing with them, and to me, that would be the objective, to have a robot that felt like you were talking to another person as opposed to a robot” (Workshop 1, Participant OA-310) | 5/7 workshops |
| Potential for damage/harm | “You definitely want it to be fast enough to get out of your way if you were, you know, walking and, yeah, I mean tripping and falling is a huge concern of seniors, and so you wouldn’t want to trip over the thing” (Workshop 1, Participant OA-305) | 6/7 workshops |
