# Supplementary material for: Older adult perspectives on emotion and stigma in social robots
Source: Front Psychiatry. 2023 Jan 12;13:1051750. doi: 10.3389/fpsyt.2022.1051750 (PMC9878396; doi:10.3389/fpsyt.2022.1051750)
Supplement: Supplementary file 8 [file Table_6.DOCX]

**Table 6.** MiRo and T-Top.

|  | **MiRo** | **T-Top** |
| --- | --- | --- |
|  | 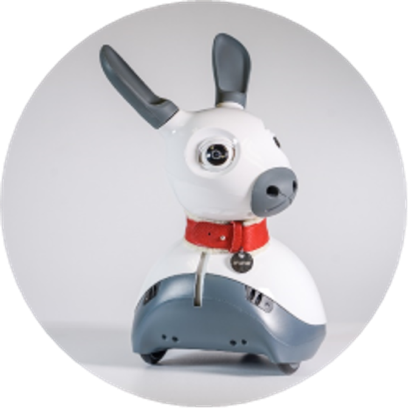 | 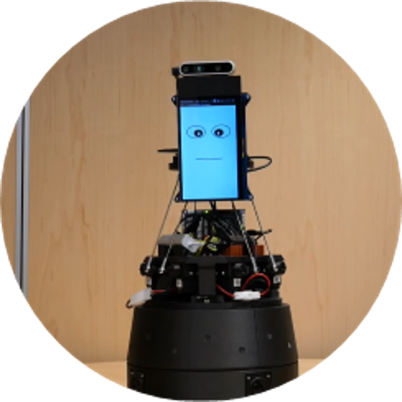 |
| Height | 30cm tall | 50cm tall |
| Appearance | Designed to look like a pet, but not one particular animal. | Has a screen that is propped up on a stand.  Display can show cartoon face or avatar. |
| Movement | Autonomous movement: detects obstacles in its way and avoids them.  Can mimic animal behaviours (e.g., tail wagging)  Moves head, eyes, and ears to perform emotion | Designed to stay in one place on a tabletop but has a rotating base and can move its ‘head’ along all axes. |
| Sounds | Non-speech sounds | Programmable human speech |
| Other key features | *Capabilities:*  Face recognition  Touch recognition  Movement recognition  *Example functionalities:*  Lights on its body that shine different colours to indicate mood (red = upset; green = happy)  Can connect to computer and be controlled from a distance | *Capabilities:*  Object and person detection  Face recognition  Voice recognition (incl. user identification)  3D room scanning  *Example functionalities:*  Performs voice-to-text  Rotates and move its “head” to follow a voice  Can ‘dance’ to a beat |
