# Supplementary material for: Older adult perspectives on emotion and stigma in social robots
Source: Front Psychiatry. 2023 Jan 12;13:1051750. doi: 10.3389/fpsyt.2022.1051750 (PMC9878396; doi:10.3389/fpsyt.2022.1051750)
Supplement: Supplementary file 9 [file Table_7.DOCX]

**Table 7.** MDRAS results.

| **Dimension** | **Statement** | **Percent agreement (%)** |
| --- | --- | --- |
| Appearance | I think a robot should have a human-like shape | 13 |
|  | I think the design of a robot should be cool | 43 |
|  | I think the design of a robot should be beautiful | 56 |
|  | I think the voice of a robot should be like the voice of a living creature | 66 |
|  | I think the shape of a robot should have roundness | 52 |
|  | I think robots should have animal-like shapes | 24 |
|  | I think the robot design should be cute | 47 |
| Self-efficacy | I can easily learn how to use a robot | 86 |
|  | It is easy to use a robot | 48 |
|  | I can make full use of a robot | 70 |
|  | I have enough skills to use a robot | 76 |
| Negative attitude | I feel scared around robots | 0 |
|  | I feel like I also become a machine when I am with a robot | 0 |
|  | It is unnatural for a robot to speak in a human language | 3 |
|  | The movements of a robot are unpleasant | 5 |
|  | It would be a pity to have a robot in my home | 0 |
| Interest | I feel easy around robots because I do not need to pay attention to robots as I do to humans | 46 |
|  | It is good if a robot can do the work of a human | 55 |
|  | Robots are neo-futuristic and cutting edge | 78 |
|  | I want to use robots if I can use them with my friends | 15 |
|  | If my friends use robots, I will also want one | 30 |
|  | If a robot is introduced to my home, I think my children or grandchildren will be pleased | 52 |
|  | I would want to boast that I have a robot in my home | 38 |
| Familiarity | I want to converse with a robot | 48 |
|  | I think a robot can be a communication partner | 58 |
|  | I like that a robot can encourage me | 61 |
|  | I would feel relaxed with a robot in my home | 70 |
|  | If a robot was introduced to my home, I would feel like I have a new family member | 19 |
