# Supplementary material for: Older adult perspectives on emotion and stigma in social robots
Source: Front Psychiatry. 2023 Jan 12;13:1051750. doi: 10.3389/fpsyt.2022.1051750 (PMC9878396; doi:10.3389/fpsyt.2022.1051750)
Supplement: Supplementary file 10 [file Table_8.DOCX]

**Table 8.** PIADS results.

| **Subscale** | **Care partner score** | | **Older adult score** | | **Person with dementia score** | |
| --- | --- | --- | --- | --- | --- | --- |
|  | **Mean** | **Standard Error** | **Mean** | **Standard Error** | **Mean** | **Standard Error** |
| Adaptability | 0.79166667 | 0.22230901 | 0.88194444 | 0.19126432 | 1.41666667 | 0.75 |
| Competence | 0.59444444 | 0.20811364 | 0.32291667 | 0.16077388 | -0.75 | NA |
| Self esteem | 0.79807692 | 0.20536595 | 0.44 | 0.14686303 | -0.125 | 1 |
