# Supplementary material for: Older adult perspectives on emotion and stigma in social robots
Source: Front Psychiatry. 2023 Jan 12;13:1051750. doi: 10.3389/fpsyt.2022.1051750 (PMC9878396; doi:10.3389/fpsyt.2022.1051750)
Supplement: Supplementary file 11 [file Table_9.DOCX]

**Table 9.** Poll results: “Would you use a social robot as a companion?”

| **Option** | **Older adults percent endorsing (%)** | **Care partners and people with dementia percent endorsing (%)** |
| --- | --- | --- |
| Yes, I would use a social robot today | 32 | 35 |
| I would not use a social robot today, but I might someday | 52 | 56 |
| I would never use a social robot now or in the future, but others might | 17 | 9 |
| I do not think anyone would use a social robot | 0 | 0 |
