# Supplementary material for: Older adult perspectives on emotion and stigma in social robots
Source: Front Psychiatry. 2023 Jan 12;13:1051750. doi: 10.3389/fpsyt.2022.1051750 (PMC9878396; doi:10.3389/fpsyt.2022.1051750)
Supplement: Supplementary file 12 [file Table_10.DOCX]

**Table 10.** Poll results: “I would feel comfortable expressing my thoughts and feelings to a social robot”

| **Option** | **Older adults percent endorsing (%)** | **Care partners and people with dementia percent endorsing (%)** |
| --- | --- | --- |
| Strongly agree | 5 | 15 |
| Agree | 61 | 47 |
| Neutral | 24 | 29 |
| Disagree | 5 | 9 |
| Strongly disagree | 6 | 0 |
